# Psychometric properties of the Maslach Burnout Inventory in healthcare professionals, Ancash Region, Peru

**DOI:** 10.12688/f1000research.139258.2

**Published:** 2024-03-05

**Authors:** Rosario Margarita Yslado Méndez, Junior Sánchez-Broncano, Carlos De La Cruz-Valdiviano, Ivette Quiñones-Anaya, Enaidy Reynosa Navarro

**Affiliations:** 1 Facultad de Ciencias Médicas, National University Santiago Antunez de Mayolo, Huaraz, Ancash, 02000, Peru; 2 Facultad de Psicología, Federico Villarreal National University, San Miguel, Lima Region, 15082, Peru; 3 Facultad de Ciencias de la Salud, Cesar Vallejo University, Trujillo, La Libertad, 13001, Peru

**Keywords:** Burnout, Psychometrics, Validity, Reliability, Healthcare Professionals

## Abstract

**Background:**

Burnout syndrome (BS) among healthcare professionals in Peru demands immediate attention. Consequently, there is a need for a validated and standardized instrument to measure and address it effectively. This study aimed to determine the psychometric properties of the Maslach Burnout Inventory (MBI) among healthcare professionals in the Ancash region of Peru.

**Methods:**

Using an instrumental design, this study included 303 subjects of both sexes (77.56% women), ranging in age from 22 to 68 years (M = 44.46, SD = 12.25), selected via purposive non-probability sampling. Appropriate content validity, internal structure validity, and item internal consistency were achieved through confirmatory factor analysis, and discriminant validity for the three dimensions was obtained. Evidence of convergent validity was found for the Emotional Exhaustion (EE) and Personal Accomplishment (PA) dimensions, with reliability values (ω > .75).

**Results:**

The EE and PA dimensions exhibited acceptable levels of reliability (ω and α > .80). However, the Depersonalization (DP) dimension demonstrated significantly lower reliability (α < .60 and ω < .50).

**Conclusions:**

A correlated three-factor model was confirmed, with most items presenting satisfactory factor loadings and inter-item correlations. Nonetheless, convergent validity was not confirmed for the DP dimension.

## Introduction

Burnout Syndrome (BS) represents a global concern in public health, as it affects health professionals with increasing frequency and intensity. BS is a disease associated with occupational risk that impacts quality of life, physical and mental health, well-being, and can even endanger life (through suicidal behaviors) of those affected by it. ^1^^,^^2^ As a result of BS, the provision of optimal patient care is hindered, as it affects job performance and productivity; the degree of satisfaction of health professionals and patients; ^3^ and it exposes healthcare facilities to economic losses and deficiencies in achieving goals. ^4^ Therefore, it's essential to periodically evaluate BS levels in healthcare staff for timely detection, treatment, and prevention of this mental and occupational health issue. This will ensure that patients receive reliable and competent clinical care.

BS emerges as a reaction to prolonged occupational stress, often surfacing when healthcare workers' stress coping mechanisms prove inadequate to handle their job-related stressors. ^5^ The Maslach Burnout Inventory (MBI) ^6^ is utilized to gauge BS, capturing three distinct dimensions: emotional exhaustion (EE), depersonalization (DP), and personal accomplishment (PA). EE signifies the sense of emotional oversaturation and fatigue from work; DP represents an impersonal and indifferent response towards patients; whereas PA indicates feelings of competency and success within the professional sphere. ^7^ An individual is diagnosed with BS when they exhibit elevated levels in the domains of EE and DP, but a diminished sense of PA.

There are multiple tools to measure BS; however, in Peru, the most commonly used instrument to assess BS levels in health professionals is the MBI. The MBI has several versions, such as the MBI-HSS, ^8^^,^^9^ the MBI-HSS (MP), ^7^ and the MBI “Burnout” Inventory by Maslach - Burnout Syndrome due to Care-related Occupational Stress, ^6^ which is a Spanish adaptation of the MBI. This adaptation is based on a three-factor structure composed of 22 items. These items are evaluated using a Likert scale ranging from 0 (never) to 6 (daily). The items are organized into three dimensions: Emotional Exhaustion (EE - 9 items), Depersonalization (DP - 5 items), and Personal Accomplishment (PA - 8 items). Subsequently, scores are categorized into BS levels: mild, moderate, and severe.

Past investigations into the psychometric characteristics of the Maslach Burnout Inventory-Human Services Survey (MBI-HSS) utilizing healthcare professional samples have yet to achieve consensus regarding the instrument's solid psychometric attributes. ^10^ Some studies confirm the 22-item three-factor structure, validity, and reliability of the MBI-HSS ^11^^,^^12^; other studies corroborate the three-factor model, but with elimination of some items. ^13^^–^^17^ Other studies have found a four-factor model ^18^ and a five-factor model. ^19^

In Peru, the three-factor structure of the MBI-HSS was confirmed in Peruvian physicians with the elimination of three items. Additionally, a three-factor model was found in Peruvian nurses with the elimination of five items. ^20^ However, there are no reports on the psychometric properties and standardization of Maslach and Jackson's MBI in its 1997 version, despite its frequent use in studies conducted in Peru. ^21^^–^^23^

## Literature review

Research pursuits have been launched worldwide, using a diverse set of statistical procedures, to scrutinize the psychometric characteristics of the MBI. These investigations have concentrated on deciphering the intrinsic structure of this measurement tool. In the context of the 22-item versions, structures ranging from one-factor, two-factor, three-factor models, ^24^ to four-factor structures ^25^ have been discerned. Some studies have advised the exclusion of certain items to augment the model fit indices. As such, one study discovered a two-factor model comprised of only 7 items ^26^; other studies recognized a three-factor model with 20 items, ^14^ 19 items, ^27^ 18 items, ^28^ or 15 items ^29^; while others detected five-factor models containing 19 items. ^30^ Lately, several authors have suggested that the bifactor model offers superior fit, enabling the entire set of indicators to load directly on a general factor, that is, the global BS and individual factors.

In 2022, González-Rodríguez *et al*. conducted a study in Spain with the goal of evaluating the psychometric properties of the MBI-HSS tool. Results indicated an acceptable fit for the four-factor correlated model (Emotional Exhaustion, Depersonalization, Personal Realization, and Interpersonal Psychological Stress). Cronbach's alpha coefficients indicated acceptable reliability for all four factors, with values between .718 and .911. ^31^

In the context of Vietnam, Thai and colleagues carried out a cross-sectional quantitative study with a sample of 1162 medical professionals, including doctors and nurses from fifteen hospitals. Their findings indicated that the 22-item version of the MBI-HSS provided a better fit to the data, effectively capturing the three interconnected facets of Burnout Syndrome, specifically Emotional Exhaustion (EE), Depersonalization (DP), and Personal Accomplishment (PA). The study also confirmed the measurement invariance of the MBI-HSS across different sexes and job roles. However, the data did not fit well for the group predisposed to common mental disorders. The conclusion drawn was that the Vietnamese version of the MBI-HSS operates as a valid measurement tool among Vietnamese professionals not at risk of mental health issues. ^12^

Also, in Iran Lin and colleagues in 2022 conducted a cross-sectional quantitative study using a sample of 306 healthcare professionals, which included 106 doctors and 200 nurses. The findings from this study indicated that the MBI-HSS-MP exhibits strong psychometric properties among healthcare professionals across all three dimensions that were examined. ^32^ Similarly, in 2021, a quantitative, exploratory, descriptive, and analytical study was carried out by Pereira and colleagues, involving a participant pool of 282 health professionals. The study confirmed the three-factor structure of the MBI. However, items 9, 12, 15, and 16 demonstrated a factor load below the suitable cut-off and were consequently omitted from the model. The results of the study validate that the MBI serves as a reliable and factorially valid instrument for evaluating burnout among healthcare professionals in Brazil. ^33^

Mukherjee *et al*., in 2022, conducted research in the United Kingdom to determine the psychometric properties of the MBI-HSS in pediatric oncology staff, analyze its factorial structure, assess its internal reliability, and evaluate whether it met the requirements of the Rasch model. They employed a quantitative approach in a sample of 203 health workers from seven major treatment centers and a charity dedicated to child cancer in the UK (comprising 115 nurses, 40 doctors, 29 social workers, and 18 play specialists) who completed the MBI-HSS. The results from the factor analysis did not support the traditional three-factor structure of the MBI-HSS. Instead, they suggested the presence of seven distinct factors. Assessments using Coefficient Alpha and Rasch modeling showed that while the Emotional Exhaustion (EE) and Personal Accomplishment (PA) subscales met the standards for interval-level measurements in group-level diagnoses, the Depersonalization (DP) subscale did not meet these benchmarks. Further investigation revealed a “floor effect” in several DP items. ^34^

Also, in 2020, a research investigation was conducted by Slabšinskienė and her team in Lithuania. Their findings confirmed that women tend to experience higher levels of emotional exhaustion compared to their male counterparts. Furthermore, it was found that dentists with more advanced specializations were significantly less prone to Burnout Syndrome than those engaged in general practice. These results not only affirm the factorial validity of the MBI but also underscore its stable structure and the differences in Burnout Syndrome dimensions across various demographic and workload sectors. ^35^

In 2020, Calderón-De la Cruz and Merino-Soto conducted a research study, the results of which revealed a three-factor internal structure within the MBI-HSS after excluding three items. The reliability was considered adequate, with scores ranging between 0.845 and 0.918. However, this dropped significantly when correlated errors were taken into account, with scores between 0.335 and 0.517. In conclusion, the suitability of the proposed 19-item structure was confirmed, validating the internal structure of the MBI-HSS among Peruvian physicians. Furthermore, the 22-item version was deemed irrelevant for the assessment of Peruvian physicians, and thus was dismissed. ^20^

In addition, in 2020 Calderón-De la Cruz and colleagues conducted a research study, the results of which identified three distinct factors. However, after excluding seven items, they produced a condensed 15-item version which exhibited satisfactory reliability, with omega coefficient values ranging between 0.797 and 0.837. This brought into question the validity of the original MBI-HSS when applied to nurses, leading them to propose this 15-item version as an alternative. In conclusion, the need to tailor the MBI-HSS to the specific needs of Peruvian nursing professionals was confirmed, as the original version was found to be unsuitable for this group. Nevertheless, a satisfactory alternative was offered in the form of a 15-item version, which demonstrated both an adequate internal structure and reliability. ^36^

Several studies have pinpointed factors related to the mental health outcomes experienced by healthcare professionals, underscoring the significant influence of workplace stressors and the importance of effective coping strategies. ^37^^,^^38^ These include: 1) insufficient hospital resources, 2) increased occupational risk due to potential virus exposure, 3) prolonged working hours, 4) irregular sleep patterns, 5) challenges in maintaining a work-life balance, 6) neglect of personal and family needs due to increased workload, and 7) inadequate communication and a lack of up-to-date information. Each of these factors has been recognized as a significant contributor to increased physical and mental fatigue, anxiety, stress, and burnout. ^39^^–^^41^

In recent years, BS has emerged as a recognized psychosocial issue stemming from prolonged occupational stress, positioning it as a critical area of focus in fields like occupational health psychology and organizational/work psychology. The ramifications of burnout include both physiological and psychological complications in employees, significantly impacting their job performance. One particular study reported a general prevalence of BS among physicians amounting to 67%, coupled with high instances of EE, DP, and diminished feelings of PA. These figures underscore the importance of ongoing research and effective interventions to manage and prevent BS among healthcare professionals and in other high-stress occupations. ^42^

In Peru, it was found that 6.9% of physicians in Arequipa presented severe levels of BS. ^43^ Likewise, a frequency of BS was found for medical interns in Lima hospitals of 33% and 35% in the years 2017 and 2018, respectively. ^44^ The occurrence of symptoms related to BS, including anxiety, depression, diminished satisfaction and quality of care, as well as post-traumatic stress and higher suicide rates, are notable concerns. ^45^ Amidst the coronavirus disease 2019 (COVID-19) pandemic, there's an intensified need to address these issues as healthcare professionals are operating within increasingly stressful environments, often encountering traumatic situations and heavy workloads. This demands a comprehensive understanding of these professionals' mental health, which is fundamental to developing effective interventions. Regular assessments, mental health support programs, coping strategies, and initiatives promoting work-life balance can all serve as crucial elements in preserving the psychological wellbeing of healthcare workers. The ultimate goal is to ensure that those who provide care are also receiving it, a consideration that has never been more critical than in the challenging context of the COVID-19 pandemic.

BS has been recognized as a health concern since it was first documented in the late 1960s. ^46^ The subsequent five decades of research, albeit uncoordinated, have produced various definitions and measures of BS worldwide, leading to some inconsistencies. The principal tool for gauging BS is the MBI, ^9^ utilized across a range of populations, including the general clinical population, ^11^ healthcare professionals with versions such as the MBI–HSS, ^31^^,^^47^ the MBI–Medical Personnel (MBI–MP), and the MBI–General Survey (MBI–GS). The original questionnaire appraises three dimensions through a total of 22 items: EE (representing feelings of being emotionally overextended by one's work), DP (a disengaged and impersonal reaction towards patients), and PA (emotions of competence and successful achievement in one's profession).

Maslach and Jackson (1981) proposed the three-dimensional model of the MBI, which includes three dimensions that together explain BS in any worker who is directly related to other people or clients. ^8^ These dimensions include: EE, which involves a feeling of being emotionally overextended and exhausted due to constant interactions with those under one's care; DP, characterized by the development of negative, detached, and cold attitudes or responses towards others; and PA, which reflects how workers evaluate themselves in relation to their work environment and professional growth. Generally, low levels of PA correspond with a perception of the work environment as being negative and failing to meet their expectations.

In terms of BS dimensions, EE often arises as a response to stress when individuals feel overwhelmed by their job requirements but lack the emotional or physical resources to cope with these demands. DP embodies the interpersonal context of work, reflecting negative or excessively detached responses towards various aspects of the job. Personal PA, on the other hand, relates to feelings of competency, efficiency, and job satisfaction. ^48^ These three dimensions have shown consistent score reliability in over 80 studies published to date. They have been further validated for human service professions, encompassing healthcare professionals. ^11^^,^^31^^,^^47^

To evaluate the psychometric properties of the MBI, researchers often use the Classical Test Theory (CTT). Within this framework, two critical concepts, reliability and validity, are fundamental for assessing the quality of a measurement tool. The understanding of validity has evolved substantially over time. According to the 1985 Standards for Educational and Psychological Testing and Manuals, it is emphasized that validity is a singular entity, where the validity of an assessment is determined by its construct validity. ^49^ The 2014 Standards argue that the term “test validity” is improper, therefore, the notion of different types of validity is considered irrelevant. Instead, these standards advocate for the provision of multifaceted information relevant to the specific purpose behind the development of the test or measurement tool. ^50^ It's important to reiterate that the validation process doesn't apply to the test itself, but rather to the interpretations drawn from individuals' scores for a specified objective.

From this perspective, the responsibility for a test's validity doesn't solely rest on the test creator, but also extends to the person administering the test. ^51^ Similarly, the validity of a test isn't definitively established, but rather involves an ongoing process of gathering evidence. ^50^ From a scientific perspective, construct validity is the only form of validity that is deemed acceptable. Therefore, the rationale and methods employed for its determination typically align with the scientific method. Evidence for construct validity is derived from multiple sources and presupposes a precise definition of the construct, along with its dimensions or facets if necessary. ^52^

Similarly, it's important to recognize that no single study validates or proves an entire theory; it only does so in relation to certain deductions that can be drawn from it. ^50^ Concerning the constructs, if the results are negative, they can be interpreted in three ways: the test may fail to measure the construct, the theoretical framework might be flawed, leading to potentially incorrect inferences, or the design of the study may not be conducive to an effective test of the hypotheses. ^53^ These interpretations suggest a deficiency in both psychometric and theoretical research knowledge, which could lead to ambiguous interpretation of negative results. Lastly, it's crucial to remember that unexpected relationships, just as much as anticipated ones, form part of the construct's nomological network and contribute to the understanding of the scores. ^52^

Alternatively, reliability is associated with the degree of consistency across multiple instances of a measurement procedure for a given group's test scores, from which the reliability and consistency of an individual's score can be inferred. ^50^ This definition implies that identical test scores should be obtained under the same administration and scoring conditions at different times. ^53^ It is also inferred that reliability refers to the precision of the measurement, independent of what the test measures. Moreover, the reliability of scores is not absolute but rather is relative to the characteristics of the group in which it is being assessed. ^54^

This study contributes to the expanding knowledge concerning the validity and reliability of scores from Maslach and Jackson's (1997) MBI for measuring burnout among healthcare professionals in Peru. ^6^ The variability in this instrument's internal structure suggests that its psychometric properties should be periodically assessed, especially when it's applied to a population where it hasn't been adapted or standardized. The practical significance of this study lies in providing a valid and reliable instrument for diagnostic assessment, clinical practice, and research, with the objective of designing prevention and intervention programs. Additionally, this study carries social relevance as it enables diagnosis of the real situation of burnout in the context of a pandemic. This helps control negative consequences and improve the mental and occupational health of healthcare professionals.

The overarching question guiding this research is: What evidence of reliability and validity does the MBI present in healthcare professionals from the Ancash region, Peru? Specific objectives are proposed:

1. Provide validity evidence of the MBI in healthcare professionals from the Ancash region, Peru, based on the content of the test through the criteria of expert judges and based on the internal structure through confirmatory factor analysis (CFA).
2. Estimate reliability of the MBI in healthcare professionals from the Ancash region, Peru, using Cronbach's alpha and omega coefficient.
3. Provide evidence of validity based on the relationship with other variables through convergent (average variance extracted, AVE) and discriminant evidence (HTMT2 ratio and the Fornell and Larcker criterion).
4. To compute MBI scores to obtain interpretative norms (reference values) for the instrument in healthcare professionals from the Ancash region, Peru, using percentile scores.

## Methods

### Study design

Applied research, which seeks to provide innovative solutions to problems that affect an individual, group, or society. ^55^ This study focused on measuring burnout in healthcare professionals from the Ancash Region, Peru. It corresponds to an instrumental design research, ^56^ as the psychometric properties of a measurement instrument will be analyzed, specifically the MBI. In this way, reliability and validity evidence will be provided for the mentioned test, as well as the establishment of interpretative norms for scores (standardization). Appendix A and B shows the first three items of this instrument.

### Population and sample

The study population included 1,844 healthcare professionals from three level II hospitals in the Ancash Region of Peru. These professionals were actively working as healthcare personnel during the COVID-19 pandemic. ^57^ The sample was collected through non-probabilistic convenience sampling. 366 participants responded to the survey, but 63 surveys were discarded due to the detection of atypical values. ^58^ Therefore, the final sample consisted of 303 participants, which allowed for a better estimation in the statistical analyses performed. The data was collected online during the months of September and October 2021. Regarding the sample size, it was estimated that, to validate this instrument, the sample should consist of at least 300 participants, as suggested by Comrey and Lee (1992). ^59^

The inclusion criteria for the study stipulated that participants must be healthcare professionals aged between 25 and 68 years, ^60^ have a minimum of one month's work experience, ^61^ and have agreed to participate in the study via informed consent. The study excluded healthcare professionals who were undergoing any psychological or psychiatric treatment, ^62^ as well as those who were on leave or had ceased their work activities during the administration of the instruments.

### Data collection, processing, and analysis

Before administering the survey, permission was sought from each director of the hospitals under investigation. Subsequently, the survey was made available only to participants who were informed and accepted the informed consent. Health professionals were invited to participate in the study via WhatsApp and emails. To avoid bias, incomplete surveys were discarded during data cleaning. The study employed an online survey method and utilized the following tools: 1) A sociodemographic form that gathered data on the following variables: age, sex, marital status, employment status, occupation, work area, type of job, care for COVID-19 patients, diagnosis with COVID-19, years of service, and hours of patient contact. 2) The Maslach Burnout Inventory (MBI) (Maslach & Jackson, 1997), which is a 22-question Likert-type questionnaire that measures feelings related to work. ^9^

The comprehensive statistical evaluation was conducted in four phases. The first phase comprised the calculation of descriptive statistics for the items, encompassing mean, standard deviation, skewness, and kurtosis. The last two coefficients represent the extent of deviation from a normal distribution, with desirable values ranging between -2 and 2. ^63^ Furthermore, the research inspected the floor and ceiling effects of the items, concentrating on the proportion of participants who selected the lowest and highest response options, respectively. Items that noted percentages equal to or less than 15% were considered devoid of these effects. ^64^ In addition, the discrimination power of the items was estimated through the item-rest corrected correlation, considering values greater than .20 as acceptable. ^65^

In the second phase, validation evidence founded on the test's internal structure was gathered via confirmatory factor analysis (CFA). The employed estimation technique was the Weighted Least Squares Mean and Variance Adjusted (WLSMV) with robust standard errors and a scaling correction (SS) statistical test, which was applied to the polychoric correlation matrix of the items. Pertaining to the goodness-of-fit indices employed to evaluate the estimated models, the ratio of chi-square to degrees of freedom (SSχ2/df) was leveraged, considering values below 2 as satisfactory. ^66^ The Comparative Fit Index (CFI) and Tucker-Lewis Index (TLI) were utilized, with values greater than .95 considered satisfactory. ^67^ The Root Mean Square Error of Approximation (RMSEA) and Standardized Root Mean Square Residual (SRMR) were also taken into account, viewing values less than .08 as acceptable. ^68^ The Weighted Root Mean Square Residual (WRMR) was included in the assessment, with values under 1 deemed satisfactory. Additionally, factor loadings that exceeded .50 were considered to be acceptable. ^66^

In the third phase, evidence of validity based on correlations with other variables was gathered. This involved evaluating both convergent and discriminant validity evidence. The assessment of convergent validity evidence was performed via the average variance extracted (AVE) for each factor, with acceptable minimum values determined according to the guidelines suggested by, ^69^ taking into account factor loadings, Cronbach's alpha reliability coefficient, and the quantity of items in the evaluated factor. Conversely, evidence of discriminant validity was derived using two methodologies. The heterotrait-monotrait ratio (HTMT) was utilized, with values below 0.85 regarded as suitable. ^70^ The Fornell-Larcker criterion was also implemented, which necessitates the comparison of the square root of the AVE for each factor with its correlations to other factors. To consider evidence of discriminant validity, the square root of the AVE for a factor should exceed its correlation with other factors. ^71^

Finally, in the fourth stage of the analysis, the dependability of test scores was evaluated using internal consistency methods. This procedure incorporated the use of the Cronbach's alpha coefficient, calculated on the basis of the covariance among the items ^72^ and omega coefficient. These coefficients range from 0 to 1, with a value of .70 or higher deemed acceptable. ^73^ In addition, MBI scores were standardized utilizing percentile scores to derive interpretive norms for the instrument.

The data analysis was carried out using R software, version 4.1.2. ^74^ A variety of packages were employed for specific functions: the tidyverse package version 1.3.0 for data manipulation, the NANIAR package version 0.6.0 for detecting missing values, the Test Data Imputation package version 1.1 for imputing missing values, the MissMech package version 1.0.2 for checking the missing completely at random (MCAR) assumption, the psych package version 2.0.8 for item analysis, the lavaan package version 0.6-7 for CFA, and the semTools package version 0.5-3 for estimating reliability, AVE, and HTMT.

In the current study, meticulous steps were undertaken to counteract and mitigate possible biases. Prior to the survey's distribution, approval was secured from the leadership of the involved hospitals. Participation was restricted to those healthcare professionals who provided their informed consent. To ensure a neutral selection process, invitations were disseminated to potential participants via WhatsApp and email. Furthermore, any incomplete responses were eliminated during the data refinement phase, safeguarding the integrity and accuracy of the concluding data.

### Ethical considerations

The General Health Law No. 26842 of Peru, ^75^ updated until the year 2022, up to date until 2022, underscores the significance of fostering scientific and health-related technological research, while also safeguarding health service providers. This study was designed to ensure no risks for the participants since it did not entail any physiological, psychological, or psychiatric alterations or interventions. Prior to the application of the study tool, informed consent was secured from the participants. The Ethics Committee of the Universidad Nacional Santiago Antúnez de Mayolo in Huaraz, Peru gave their approval for the study via REPORT No. 0010-2022-UNASAM-DII/CEI/M, on July 17, 2021.

## Results

Before conducting the statistical analysis, the initial data were thoroughly reviewed to identify missing data and outliers. In this regard, no missing values were found, but 26 univariate outliers were detected (7 in DP and 19 in PA) using the absolute deviation from the median. Additionally, 37 multivariate outliers were observed through a robust version of Mahalanobis distance. ^58^ Therefore, 63 cases were removed to obtain a final database consisting of 303 participants, which allowed for a better estimation in the conducted statistical analyses. ^88^ It's crucial to mention that the sample size achieved in this study surpasses the suggested minimum of 300 cases for studies utilizing factor analysis. ^76^

Table 1 provides a detailed overview of the demographic data of the sample population. The healthcare professionals participating in the study had ages ranging from 22 to 68 years, with an average age of 44.46 and a standard deviation of 12.25. The sample was largely made up of females, representing 77.56% of the total. In terms of marital status, those who are married constituted the largest proportion, at 43.23%. The majority of participants were employed in permanent positions (62.71%). Regarding occupation, the majority were nurses (38.94%) and belonged to various areas such as gynecology-obstetrics (15.84%), Covid-19 (13.86%), and emergency (13.53%). Additionally, most participants worked in on-site settings (88.45%). Regarding questions related to Covid-19, the majority of healthcare professionals did not have direct contact with confirmed Covid-19 patients (55.12%) and were not diagnosed with a Covid-19 infection themselves (66.34%). Finally, the majority of the sample had a tenure of 1 to 5 years (37.29%) and had more than 7 hours of patient contact per day (70.96%).

**Table 1.** Participant sociodemographic characteristics.

Variable	Category	n	%
Age	From 22 to 29	27	8.91
	From 30 to 39	107	35.31
	From 40 to 49	59	19.47
	50 to 68	110	36.30
Sex	Female	235	77.56
	Male	68	22.44
Marital status	Married	131	43.23
	Cohabiting	40	13.20
	Divorced	9	2.97
	Separated	17	5.61
	Single	103	33.99
	Widowed	3	0.99
Employment status	Employed	113	37.29
	Permanent employee	190	62.71
Occupation	Social worker	13	4.29
	Nurse	118	38.94
	Doctor	54	17.82
	Nutritionist	7	2.31
	Obstetrician	33	10.89
	Dentist	5	1.65
	Psychologist	3	0.99
	Pharmacist	2	0.66
	Nursing technician	54	17.82
	Laboratory technician	4	1.32
	Medical technologist	10	3.30
Work area	Surgery	17	5.61
	COVID-19	42	13.86
	Emergency	41	13.53
	Gynecology-obstetrics	48	15.84
	Department of Medicine	16	5.28
	Pediatrics	20	6.60
	Intensive Care Unit (ICU)	13	4.29
	Others	106	34.98
Type of work	In-person modality	268	88.45
	Remote modality	30	9.90
	Semi-presential	5	1.65
Care for patients with COVID-19	No	167	55.12
	Yes	136	44.88
Diagnosed with COVID-19	No	201	66.34
	Yes	102	33.66
Length of service	From 1 to 5 years	113	37.29
	From 6 to 10 years	70	23.10
	From 11 to 20 years	42	13.86
	From 21 years and older	78	25.74
Hours of patient contact	None	15	4.95
	From 1 to 6 hours	73	24.09
	From 7 hours and more	215	70.96

Table 2 presents the descriptive and discrimination analysis of the items. Regarding the central tendency of the items, item BS_19 obtained the highest average score (M = 5.43), while the lowest mean was found in item BS_22 (M = 0.68). In terms of item dispersion, item BS_06 had the highest variability (SD = 1.92), while the lowest variability was observed in item BS_19 (SD = 0.93). Regarding the item shape measures, the skewness and kurtosis values indicated that items BS_04, BS_09, BS_12, BS_19, and BS_22 had issues in both indicators (outside the range of -2 and 2). Additionally, items BS_05, BS_07, BS_13, BS_17, and BS_18 showed excessive kurtosis (values greater than 2). These results suggest that, in the mentioned items, the distributions do not follow a normal curve. Furthermore, most of the items exhibited floor and ceiling effects, except for items BS_01 and BS_02. Finally, the corrected item-test correlation indicated that the majority of the items had good discriminative ability, as they obtained values greater than .20, except for items BS_05, BS_10, BS_15, and BS_22, which belong to the dimension of DP.

**Table 2.** Descriptive statistics and discrimination of items.

						Effect (%)	
Item	*M*	*SD*	*Sk*	*Ku*	*ritc*	Floor	Ceiling
Emotional exhaustion							
BS_01	2.52	1.70	0.18	-1.10	.670	12.54	1.98
BS_02	3.28	1.73	-0.19	-1.16	.631	4.29	7.92
BS_03	1.81	1.68	0.69	-0.56	.652	28.05	1.98
BS_06	1.85	1.92	0.81	-0.59	.555	33.00	6.27
BS_08	2.11	1.69	0.41	-1.13	.755	18.48	0.33
BS_13	0.90	1.35	1.79	2.65	.421	53.80	0.66
BS_14	1.97	1.74	0.59	-0.82	.559	24.09	2.64
BS_16	1.42	1.46	1.08	0.64	.438	33.00	1.98
BS_20	1.84	1.72	0.67	-0.68	.678	28.38	2.31
Depersonalization							
BS_05	0.81	1.22	1.72	2.67	.132	57.76	0.33
BS_10	1.16	1.82	1.66	1.57	.147	56.44	7.26
BS_11	1.27	1.72	1.32	0.67	.209	48.84	3.63
BS_15	1.00	1.88	1.81	1.73	.052	68.65	7.26
BS_22	0.68	1.19	2.37	6.15	.186	62.71	1.32
Personal Accomplishment							
BS_04	5.30	1.32	-2.22	4.45	.239	0.99	67.66
BS_07	5.13	1.33	-1.90	3.38	.322	1.32	55.78
BS_09	5.33	1.23	-2.39	5.87	.380	1.32	64.69
BS_12	5.35	1.06	-2.34	6.73	.353	0.66	59.74
BS_17	5.04	1.27	-1.56	2.07	.420	0.33	48.51
BS_18	5.08	1.25	-1.57	2.44	.405	0.99	52.15
BS_19	5.43	0.93	-2.12	5.11	.490	0.00	62.71
BS_21	4.80	1.42	-1.19	0.52	.338	0.33	40.92

Table 3 exhibits how validity evidence, predicated on the content of the MBI, recognized as one of the five key sources of validity evidence ^50^ was gathered during this study. This evidence was assembled via the assessment performed by six specialists versed in areas such as quantitative methodology, psychometrics, instrument design, and mental health metrics. These experts appraised the relevance, representativeness, and lucidity of the items using a five-point scale (1 = not at all to 5 = entirely), as well as their essentiality for assessing burnout in healthcare professionals. A majority of these expert evaluators were clinical psychologists with over eight years of professional service, and their ages ranged between 35 to 60 years.

**Table 3.** Content-based validity evidence of the test.

	Relevance		Representativeness		Clarity		
Item	* *M*	** *V*	*M*	*V*	*M*	*V*	***CVI
Emotional exhaustion							
BS_01	4.67	.92	5.00	1.00	5.00	1.00	1.00
BS_02	5.00	1.00	4.83	.96	4.67	.92	1.00
BS_03	4.33	.83	4.67	.92	4.83	.96	1.00
BS_06	4.83	.96	4.83	.96	5.00	1.00	1.00
BS_08	5.00	1.00	4.83	.96	5.00	1.00	1.00
BS_13	5.00	1.00	4.83	.96	4.83	.96	1.00
BS_14	4.67	.92	4.67	.92	5.00	1.00	1.00
BS_16	4.67	.92	4.67	.92	5.00	1.00	1.00
BS_20	5.00	1.00	4.83	.96	4.83	.96	1.00
Depersonalization							
BS_05	4.83	.96	4.83	.96	5.00	1.00	.67
BS_10	4.67	.92	4.67	.92	5.00	1.00	1.00
BS_11	4.33	.93	5.00	1.00	4.67	.92	1.00
BS_15	5.00	1.00	4.67	.92	4.83	.96	1.00
BS_22	4.83	.96	4.83	.96	4.83	.96	.33
Personal Accomplishment							
BS_04	4.67	.92	4.83	.96	4.83	.96	1.00
BS_07	4.83	.96	4.83	.96	4.83	.96	1.00
BS_09	4.83	.96	4.83	.96	4.83	.96	1.00
BS_12	4.50	.88	4.83	.96	4.67	.92	1.00
BS_17	4.83	.96	5.00	1.00	4.83	.96	1.00
BS_18	5.00	1.00	5.00	1.00	4.83	.96	1.00
BS_19	4.50	.88	4.67	.92	5.00	1.00	1.00
BS_21	4.67	.92	4.67	.92	4.83	.96	1.00

* M = Mean.

** V = V de Aiken.

*** CVI = Lawshe's Content Validity Index.

The Aiken's V coefficient, which spans from 0 to 1, was employed to acquire a quantitative measurement of the judges' evaluation of the items' relevance, representativeness, and clarity, with a value nearing 1 suggesting a more favorable evaluation of the item. In the context of this study, values greater than .70 were deemed sufficient. ^77^ In order to quantitatively encapsulate the assessment of whether the items were indispensable for measuring BS or not, the Content Validity Ratio (CVR) was used, adopting the method suggested by. ^78^ An item was regarded as essential if it had a CVR of 1.00, contingent on the quantity of judges. ^79^

Regarding the results of the 22 items in the instrument ( Table 3), all of them had Aiken's V values above .70 in the evaluation of item content. This indicates that the items are relevant, representative, and clear for measuring the target variable. ^77^ In terms of the assessment of whether the items are essential or not, most of them showed critical values equal to 1.00, except for items BS_05 and BS_22, which had values of .67 and .33, respectively. Therefore, the six judges evaluated items BS_05 and BS_22 as non-essential for measuring burnout in healthcare professionals. Both items belong to the dimension of DP.

Table 4 shows the validity tests in terms of internal structure by confirmatory factor analysis. Four models, as identified in previous literature, were scrutinized: an oblique three-factor model, a hierarchical three-factor model inclusive of a general factor, a unifactorial model featuring a solitary general factor, and a bifactorial model that concurrently tests both a three-factor unrelated model and a unifactorial model. The first two models showed good fit indices (SSχ2/gl < 3, RMSEA < .80, CFI > .90, TLI > .90, SRMR < .80, and WRMR close to 1). However, the unifactorial model had inadequate fit indices ( Table 4), and the bifactorial model did not converge, so both models were discarded.

**Table 4.** Goodness-of-fit indices for three confirmatory factor analysis models.

Model	SSχ2	gl	SSχ2/gl	RMSEA [IC 95%]	CFI	TLI	SRMR	WRMR
Oblique	524.92	206	2.55	.072 [.064, .079]	.937	.929	.075	1.152
Hierarchical	524.92	206	2.55	.072 [.064, .079]	.937	.929	.075	1.152
Unifactorial	1215.56	231	5.26	.126 [.119, .133]	.800	.779	.122	1.912

Both the oblique and hierarchical models displayed comparable values in their goodness-of-fit indices. Therefore, to discern which model was superior, the reliability of the score values was examined across both models. In the hierarchical model, the reliability of the general factor was very low (ω = .483), so this model was discarded from the study.

**Figure 1.**
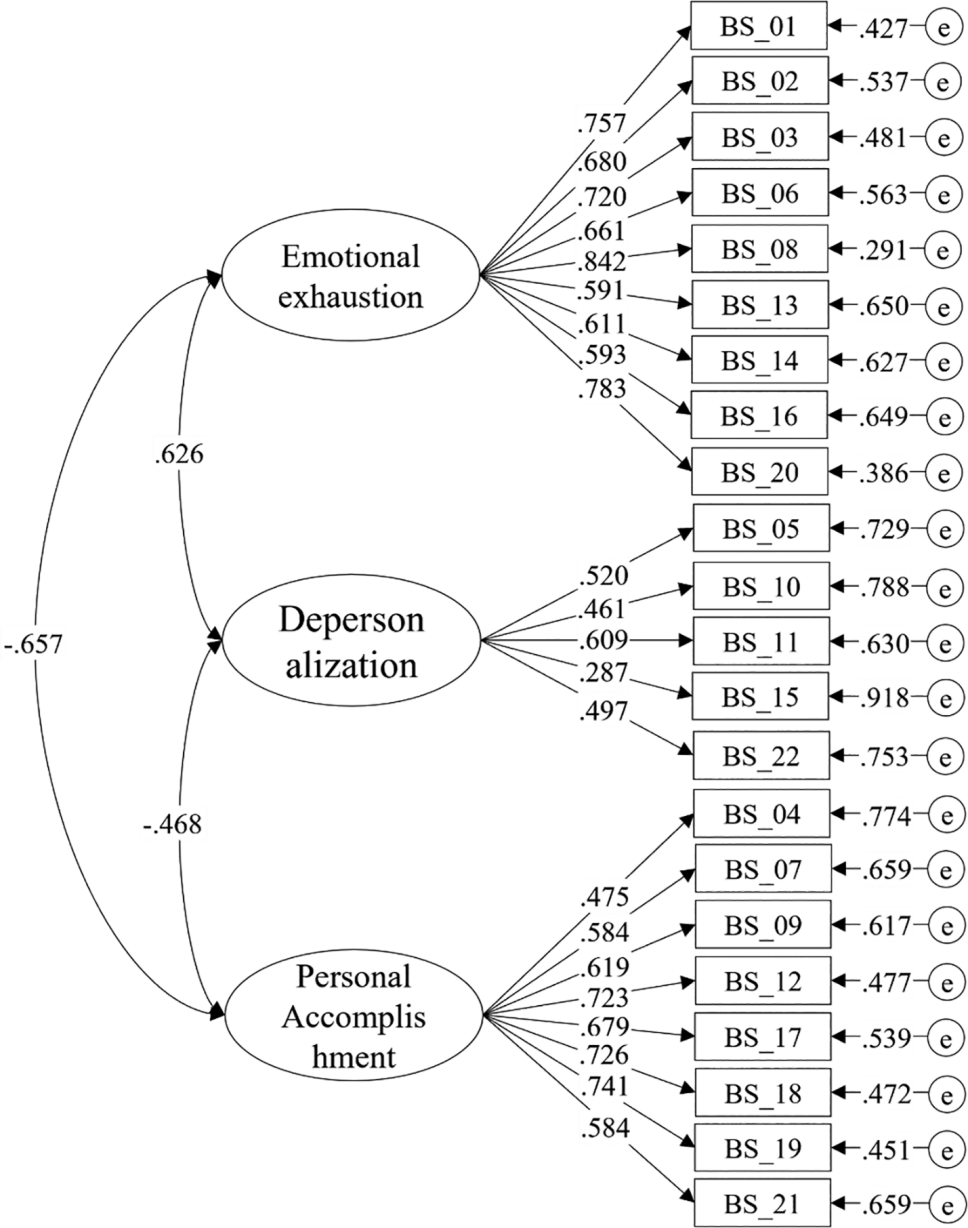
Correlated three-factor model of the Maslach Burnout Inventory (MBI).

Consequently, the correlated three-factor model was analyzed, as shown in Figure 1. Most items in this model possessed factor loadings greater than .40, with the exception of item BS_15, which had a factor loading of .287. This same item also performed poorly at the descriptive level with the lowest discrimination ( *ritc* = .052) and the highest floor effect (68.65%) compared to the other items. Regarding the interrelation among the factors, significant and moderate correlation values were noted. The correlation between EE and DP stood at .626, between EE and PA was -.657, whereas the correlation between DP and PA was -.468, as depicted in Figure 1.

As shown in Table 5, the reliability of MBI scores was assessed via the internal consistency of the items, employing alpha (α) and omega (ω) coefficients, given that the measurement model was tau-equivalent, as verified through confirmatory factor analysis (χ2 = 628.190, df = 225, χ2/df = 2.79, SRMR = .093, RMSEA = .077 [95% CI .070, .084], CFI = .920, TLI = .918). The results inferred that the dimensions of EE and PA exhibited satisfactory levels of reliability (with ω and α values greater than .80). However, in the dimension of DP, reliability was very low (α < .60 and ω < .50). Furthermore, the strength of correlations between items within each dimension was scrutinized. The analysis revealed that within the dimensions of EE and PA accomplishment, the average inter-item correlation surpassed .40. However, within the DP dimension, the items exhibited a weaker correlation to each other, yielding an average inter-item correlation of just .21.

**Table 5.** Inter-item correlation, evidence of discriminant validity, and reliability.

		Inter-item correlation							
Variable	n	Min	Max	*M*	*SD*	*AVE*	α	ω	95% CI
Emotional Exhaustion	9	.28	.69	.46	.11	.488	.886	.883	.850 – .903
Depersonalization	5	.07	.41	.21	.09	.237	.574	.492	.378 – .578
Personal Accomplishment	8	.22	.55	.41	.08	.419	.848	.802	.760 – .833

Concerning the evidence of convergent validity, assessed via the Average Variance Extracted (AVE), an acceptable level was detected for the dimensions of emotional exhaustion and personal accomplishment, this being substantiated by their reliability values (with ω values exceeding .75), number of items (between 7 and 9), and magnitudes of the factor loadings of their items, which were above .50. ^69^ However, in the dimension of DP, the AVE was lower than .25, indicating that this dimension lacks evidence of convergent validity or variance attributable to the specified measurement model.

Pertaining to the evidence of discriminant validity, all three dimensions demonstrated suitable values for the HTMT2 ratio (below .85), recording values of .686 (emotional exhaustion), .306 (depersonalization), and .678 (personal accomplishment). However, the Fornell and Larcker criterion was solely satisfied by the EE dimension, as its square root of the AVE (.699) surpassed the correlations with the other two factors. ^71^

Table 6 presents percentile norms for the three dimensions of the MBI, with three categories (low, medium, and high) corresponding to the 25th, 50th, and 75th quartiles, respectively. ^80^ For the dimension of EE, scores between 0 and 9 indicate a low level, between 10 and 24 indicate a medium level, and scores between 25 and 54 indicate a high level. In the dimension of DP, scores between 0 and 1 indicate a low level, between 2 and 7 indicate a medium level, and scores between 8 and 30 indicate a high level. For the dimension of PA, scores between 0 and 39 correspond to a low level, between 40 and 45 indicate a medium level, and scores between 46 and 48 indicate a high level. Considering these factors, elevated scores in EE and DP, coupled with low scores in personal accomplishment, are deemed suggestive of BS issues.

**Table 6.** Percentile norms for the dimensions of the MBI.

Percentile	Emotional exhaustion	Depersonalization	Personal Accomplishment	Category
99	37–54	14–30	–	High
95	34–36	11–13	–	
90	32–33	10	48	
85	28–31	–	–	
80	26–27	9	47	
75	25	8	46	
70	23–24	7	–	Average
65	20–22	6	44–45	
60	19	–	–	
55	17–18	5	43	
50	16	4	42	
45	14–15	–	–	
40	13	3	41	
35	12	2	40	
30	10–11	–	–	
25	9	1	38–39	Low
20	8	–	36–37	
15	6–7	0	34–35	
10	4–5	–	32–33	
05	1–3	–	30–31	
01	0	–	0–29	
M	17.70	4.91	41.46	
DE	10.45	4.07	5.43	
Skewness	0.39	0.56	-0.68	
Kurtosis	-0.66	-0.63	-0.48	

## Discussion

The results indicated that most items of the MBI showed good discriminative ability within their respective dimensions, except for four items in the DP dimension, indicating low discrimination. Regarding the validity of the instrument, evidence based on content showed that the items were relevant, representative, and clear for measuring burnout, although two DP items were assessed as non-essential. In addition, the confirmatory factor analysis revealed a good fit for the three-factor correlated model. Convergent evidence was found within the dimensions of PA and EE, while discriminant evidence was present across all three dimensions. Finally, percentile norms were created for interpreting scores. It was determined that the EE and PA dimensions displayed satisfactory internal consistency, whereas the DP dimension demonstrated relatively low reliability.

Descriptive item analysis mainly indicated kurtosis problems, meaning that a high percentage of participants chose only one response option, resulting in floor or ceiling effects. In some cases, these effects were excessive, such as in items BS_13, BS_05, BS_10, BS_15, and BS_22, where more than 50% of respondents chose the lowest alternative (floor effect), with the latter four items belonging to the DP dimension, suggesting a general tendency to have low scores in that dimension. On the other hand, the most notable ceiling effect was observed in items BS_04, BS_07, BS_09, BS_12, BS_18, and BS_19 (all related to PA), as more than 50% of the sample selected the highest response option, indicating that the majority of participants had a tendency to obtain high scores in that dimension.

Regarding the discrimination of items within each dimension, assessed through corrected item-test correlations, it was found that most items exceeded the criterion of .20, indicating good discriminative ability. However, four items in the DP dimension (BS_05, BS_10, BS_15, and BS_22) showed low values, ranging from .052 to .186, suggesting a weak association of each item with the overall score in that specific dimension. Additionally, the remaining DP item (BS_11) slightly surpassed the threshold of .20 ( *r _itc_* = .209). As a result, the DP items demonstrated poor discriminative ability.

These results differ from the study by, ^81^ where all items showed a corrected item-test correlation greater than .20, as well as from the study by González-Rodríguez *et al*. (2022), where only item BS_04 had a value below the indicated criterion ( *r _itc_* = .114), and from the research by Oh and Lee (2009), where item BS_14 was the only one that exhibited low discrimination ( *r _itc_* = .030). ^31^ The difference in results could be attributed to the sample used, as the mentioned studies worked with participants who were not healthcare professionals (e.g., university teachers). Additionally, there are also differences in the calculation of the corrected item-test correlation, as ^81^ y ^31^ correlated the items with the total MBI score, while in the present study, the analysis was conducted within each dimension, similar to what ^82^ did.

Regarding the validity analysis, three sources of evidence were collected: content-based evidence, internal structure evidence, and evidence of relationships with other variables. Evidence for content-based validity was gathered from the assessments made by six expert judges who evaluated the relevance, representativeness, clarity, and essentiality of the items for measuring burnout. The assessments made by the judges were positive in regard to relevance, representativeness, and clarity for all items. The Aiken's V coefficient was employed to compile their responses, resulting in values above .70 (with a range from .83 to 1.00). These findings complement the results of, ^81^ who also assessed general aspects of the MBI based on the judgment of eight experts, with Aiken's V coefficients ranging from .88 to 1.00. On the other hand, the judges indicated that almost all items were essential, except for BS_05 and BS_22, which were considered non-essential for measuring BS, with Lawshe's validity index values of .67 and .33, respectively. Both items belong to the DP dimension.

Concerning the evidence for validity based on the internal structure, four models were evaluated using confirmatory factor analysis. Both the unidimensional model and the bifactor model didn't produce satisfactory results. The unidimensional model showed insufficient goodness-of-fit indices, and the bifactor model failed to achieve computational convergence, possibly due to the complexity of the bifactor model coupled with the sample size. In the bifactor model, convergence with subsequent modifications (e.g. deletion of items) was not assessed to avoid capitalisation of chance and possible under-representation of dimension content. The last two models, the three-factor correlated model (comprising EE, DP, and PA) and the hierarchical model (which includes the three aforementioned factors and an overarching general factor - BS - underlying them), both obtained adequate and equivalent fit indices.

In the hierarchical model, despite having good fit indices (SS *χ2/gl* < 3, RMSEA < .80, CFI > .90, TLI > .90, SRMR < .80, and WRMR close to 1), the general factor of burnout showed low internal consistency (ω = .483), indicating low reliability of the scores. Therefore, the hierarchical model was discarded, and only the three-factor correlated model was interpreted. This model aligns with the findings of Loera *et al*. (2014) in a group of Italian nurses, although in that study, items BS_12 and BS_16 were excluded from the model. It also aligns with the study by ^28^ in Finnish nurses, although items BS_6, BS_13, BS_16, and BS_22, as well as the correlation between the errors of items BS_17 and BS_18, were excluded. Additionally, ^83^ found the same structure in Peruvian nurses, both with the full 22-item version of the MBI and with a reduced 15-item version. Similar findings were also found by ^83^ in a sample of nursing staff from public health institutions in Peru, using the general 16-item version (MBI-GS).

Several studies have echoed the findings of the current research, identifying the same three-factor correlated structure, although these studies have been conducted in domains beyond healthcare. For instance, a study in South Korea identified the same three dimensions within a cohort of child protection service workers, however, a condensed 15-item variant displayed superior goodness-of-fit indices during the confirmatory factor analysis. ^82^ Nonetheless, there are also studies that present varying and contradictory structures. For example, consulted studies highlight discovered a four-factor model in a sample of Spanish social workers, where the fourth factor was designated as interpersonal psychological strain. ^31^ This result, however, was achieved through a principal component analysis, selecting the quantity of factors according to Kaiser's rule of eigenvalues exceeding 1, and employing Varimax rotation. These practices are currently dissuaded as they may yield incorrect and biased estimations. ^76^^,^^84^

Within the CFA, as well as in the descriptive analyses, item BS_15 was the most problematic item with the lowest factor loading (λ = .287). The content of the item “I really don't care about what happens to some of my patients” refers to the lack of concern on the part of health care staff about the health of their patients. In the pandemic context where the study was conducted, COVID-19 was a collective problem, so it is natural that there is a direct or indirect concern for patients. This could be one of the reasons why the item did not work well in the model and why it did in pre-pandemic studies. One reason from a methodological point of view is that it is the only item in the whole PD dimension, and the whole test, that contains an explicit negation (not), which may have given problems in the comprehension of the item when it was answered. Despite the above, it was decided to keep the item, as its content is not interchangeable with any other item in the PD dimension and removing it would imply an under-representation of content in the dimension. Moreover, it would limit the possibility of directly comparing the results obtained here with previous studies.

Regarding evidence of validity based on correlation with other variables, both the EE and PA displayed both convergent and discriminant validity. Conversely, the DP dimension solely exhibited evidence of discriminant validity. Convergent validity evidence was obtained via the Average Variance Extracted (AVE), which encompassed an exhaustive analysis of this indicator in conjunction with other elements such as reliability, factor loadings, and the count of items. ^69^ The dimension of depersonalization exhibited an unacceptable level (AVE = .237) and thus did not meet this validity criterion.

Furthermore, two methods were employed to assess discriminant validity: the HTMT2 ratio and the criterion by. ^71^ The results were consistent for the dimension of EE, indicating that it possesses discriminant validity evidence. However, for the other two dimensions, while the HTMT2 ratio was adequate, the results differed according to the. ^71^ This discrepancy arose as the square root of the AVE for both dimensions was lower than their correlations with another factor.

Despite the criterion by ^71^ not being acceptable for DP and PA, it was decided to give greater importance to the results of the HTMT2, as this criterion is better suited for discriminant validity evidence within the framework of structural equation models. ^70^ In light of this, all three dimensions showcase discriminant evidence, signifying that EE, DP, and PA are distinct from each other in their measurement of BS. In simpler terms, they encapsulate different facets of BS in the manner this variable is quantified using the MBI.

Regarding the reliability of scores in the correlated three-factor model, both EE and PA exhibited acceptable levels of internal consistency (ω and α > .80). These findings are consistent with what has been reported in other studies, where a ω value above .70 was found ^24^^,^^36^^,^^81^^,^^85^^,^^86^ and a value of α above .70 ^28^^,^^29^^,^^36^^,^^81^^,^^85^ for these dimensions.

However, the dimension of DP displayed low levels of internal consistency (α = .574 and ω = .492). These reliability results for this dimension have also been found in previous studies. ^20^^,^^29^^,^^81^ Therefore, the DP scores demonstrate low reliability. This is consistent with the previous results presented for this dimension, as four items exhibited low discriminant ability, two items were considered non-essential by expert judges, one item had the lowest factor loading in the confirmatory factor analysis (less than .40), the items had lower inter-item relationships, with an average inter-item correlation of .21. Additionally, it was the only factor that did not have evidence of convergent validity.

Lastly, percentile norms were established for interpreting scores on the MBI among healthcare professionals in the Ancash Region. Three categories were delineated in this regard: low, moderate, and high, corresponding to the first quartile (25%), second quartile (50%), and third quartile (75%) respectively. ^80^ However, it was suggested to apply the interpretational meaning for the dimensions of EE and PA, but not for the DP dimension, due to its observed low level of reliability and other unsatisfactory indicators discussed in this section. Additionally, the calculation of a total BS score was not feasible based on the study's findings, as the general factor observed in the hierarchical model also showed weak internal consistency. Thus, based on the aforementioned, it was inferred that an individual is likely to experience burnout problems if they present a high score in EE and a low score in PA.

One of the study's limitations was the size of the sample utilized. Initially, there were 366 participants, but after removing univariate and multivariate outliers, the final sample was reduced to 303 cases, limiting the ability to perform further statistical analyses such as factorial invariance or item differential functioning analysis. However, the sample size was still larger than the recommended minimum of 300 cases for psychometric studies when the number of items is not very large. ^54^^,^^76^ Moreover, the study examined convergent and discriminant validity evidence based on the factors of the MBI, without incorporating any external variables beyond BS (for instance, using additional instruments). Nonetheless, the study did utilize indicators that are currently recommended for such an analysis, like AVE (Average Variance Extracted) and the HTMT2 ratio. ^69^^,^^70^

### Strength

The main contribution of this study is that it goes beyond psychometric properties by establishing percentile standards for the MBI dimensions and their respective low, medium, and high categories. This facilitates the interpretation of the test scores (these standards will enable the interpretation of results in future applications). The direct scores obtained by a participant in the test, on their own, are meaningless; to interpret them, it's essential to refer to established standards. Therefore, the instrument can be used in clinical, organizational, and research areas for the design of public policies aimed at the prevention and intervention of BS. Furthermore, Ancash is a region in Peru where healthcare professionals working in its 12 hospitals exhibit higher levels of BS compared to healthcare staff from other regions. ^21^^–^^23^^,^^87^ As such, this at-risk group should be evaluated semi-annually or annually with an objective, valid, and reliable instrument, one that offers quick and practical scoring, like the one presented in this study.

### Limitations

Firstly, while the sample size used exceeded the minimum recommendation of 300 cases for psychometric studies, ^54^^,^^56^^,^^76^ it restricted our ability to conduct further statistical analyses, such as factorial invariance or differential item functioning analysis. Secondly, the study's focus on convergent and discriminant validity was solely based on MBI factors, without introducing any external variables beyond BS. For instance, no additional instruments were utilized. Nonetheless, the study employed currently recommended indicators for such analyses, like AVE (Average Variance Extracted) and the HTMT2 ratio. ^69^^,^^70^ Lastly, while a non-probabilistic convenience sampling was theoretically justified and used, caution is advised when replicating this study in different settings or in follow-up research. Additionally, it is suggested that future studies be conducted on larger probabilistic samples and include other types of validity and reliability to enhance and standardize the instrument.

## Conclusions

The MBI proves to be an effective tool for assessing burnout in healthcare professionals, particularly in terms of its dimensions of EE and PA. It demonstrates evidence of content validity, internal structure (confirming a correlated three-factor model), and relationships with other variables (both convergent and discriminant evidence). Additionally, it showcases its reliability through the internal consistency of scores. However, the use of DP scores is not recommended due to their low reliability, with item BS_15 exhibiting a low factor loading (.287). Therefore, this psychometric instrument can be utilized to measure BS in research studies where this variable is involved, whether it is to describe its prevalence in a specific group, compare scores across different groups, or understand its relationship with other variables that are part of its nomological network.

## Data Availability

Zenodo: Psychometric Properties of the MBI in Healthcare Professionals, Ancash Region, Peru. https://doi.org/10.5281/zenodo.8048477. ^88^ This project contains the following underlying data:
- Database (Statistical results after the application of the Maslach Burnout Inventory).xlsx - Final results (PDF).docx Zenodo: Psychometric Properties of the MBI in Healthcare Professionals, Ancash Region, Peru. https://doi.org/10.5281/zenodo.8048477. ^88^ This project contains the following extended data:
- Data key for the instrument.pdf - The Aiken's V.xlsx - The Lawshe's CVR.xlsx Data are available under the terms of the Creative Commons Attribution 4.0 International license (CC-BY 4.0).
