# Supplementary material for: Psychometric properties of the Maslach Burnout Inventory in healthcare professionals, Ancash Region, Peru
Source: F1000Res. 2024 Mar 5;12:1253. Originally published 2023 Oct 2. [Version 2] doi: 10.12688/f1000research.139258.2 (PMC10925071; doi:10.12688/f1000research.139258.2)
Supplement: Peer review history

# Reviewer report: Reviewer response for version 2

**DOI:** 10.5256/f1000research.162984.r252923

**Published:** 2024-04-15

**Authors:** Arnold Alejandro Tafur Mendoza

**Affiliations:** 1 Universidad Peruana de Ciencias Aplicadas, Lima District, Lima Region, Peru

Approved.

Is the work clearly and accurately presented and does it cite the current literature?

Yes

If applicable, is the statistical analysis and its interpretation appropriate?

Yes

Are all the source data underlying the results available to ensure full reproducibility?

Yes

Is the study design appropriate and is the work technically sound?

Yes

Are the conclusions drawn adequately supported by the results?

Yes

Are sufficient details of methods and analysis provided to allow replication by others?

Yes

Reviewer Expertise:

psychometrics, measurement, assessment, quantitative methodology, factor analysis.

I confirm that I have read this submission and believe that I have an appropriate level of expertise to confirm that it is of an acceptable scientific standard.

# Reviewer report: Reviewer response for version 2

**DOI:** 10.5256/f1000research.162984.r252924

**Published:** 2024-03-09

**Authors:** Brian Norman Peña Calero

**Affiliations:** 1 Universidad Peruana de Ciencias Aplicadas, Lima District, Lima Region, Peru

Everything looks good. No additional comments.

Is the work clearly and accurately presented and does it cite the current literature?

Yes

If applicable, is the statistical analysis and its interpretation appropriate?

Yes

Are all the source data underlying the results available to ensure full reproducibility?

Yes

Is the study design appropriate and is the work technically sound?

Yes

Are the conclusions drawn adequately supported by the results?

Partly

Are sufficient details of methods and analysis provided to allow replication by others?

Yes

Reviewer Expertise:

Specialist in data and psychometric analysis, research development and research and analysis consultancy.

I confirm that I have read this submission and believe that I have an appropriate level of expertise to confirm that it is of an acceptable scientific standard.

# Reviewer report: Reviewer response for version 1

**DOI:** 10.5256/f1000research.152521.r211342

**Published:** 2024-01-30

**Authors:** Brian Norman Peña Calero

**Affiliations:** 1 Universidad Peruana de Ciencias Aplicadas, Lima District, Lima Region, Peru

Although the psychometric results of the instrument are good, it would be convenient to add the revision of an additional model without item 15 and any other item that impairs the estimation of its reliability. Additionally, if a second-order model is suspected, it would be convenient to include the assessment of a bifactor model.

 The article appropriately evaluates the psychometric behavior of the Maslach Burnout Inventory (MBI), highlighting the participant selection and the psychometric analysis plan to achieve research objectives. To improve the communication of the results, the following suggestions are made:

1. In Table 5, where a low factorial load for item 15 is indicated, it could be added that this item also had inadequate performance in the descriptive analysis (Table 2).
2. In the discussion section, it is recommended to detail more about the poor performance of item 15, its content, and possible reasons for its inadequacy in the model.
3. If deciding to keep item 15 in the model, despite its significant difference in factorial loads, explaining the reason would be advisable. Otherwise, evaluating the depersonalization factor without this item could be considered.
4. Regarding the bifactor model, it would be useful to mention if its convergence was evaluated with subsequent modifications, such as the removal of items.

With these minor modifications, the article will be able to provide significant insight into the psychometric behavior of the instrument of interest.

Is the work clearly and accurately presented and does it cite the current literature?

Yes

If applicable, is the statistical analysis and its interpretation appropriate?

Yes

Are all the source data underlying the results available to ensure full reproducibility?

Yes

Is the study design appropriate and is the work technically sound?

Yes

Are the conclusions drawn adequately supported by the results?

Partly

Are sufficient details of methods and analysis provided to allow replication by others?

Yes

Reviewer Expertise:

Specialist in data and psychometric analysis, research development and research and analysis consultancy.

I confirm that I have read this submission and believe that I have an appropriate level of expertise to confirm that it is of an acceptable scientific standard, however I have significant reservations, as outlined above.

# Response

**Published:** 2024-02-20

**Authors:** Enaidy Reynosa Navarro

**Affiliations:** Universidad César Vallejo, Peru

Observation 1: The suggestion was added to the manuscript: *“This same item also performed poorly at the descriptive level with the lowest discrimination (ritc = .052) and the highest floor effect (68.65%) compared to the other items.”*

 Observation 2: The suggestion was added to the manuscript: *“Within the CFA, as well as in the descriptive analyses, item BS_15 was the most problematic item with the lowest factor loading (λ = .287). The content of the item "I don't care about what happens to some of my patients" refers to the lack of concern on the part of healthcare staff about the health of their patients. In the pandemic context where the study was conducted, COVID-19 was a collective problem, so, naturally, there is a direct or indirect concern for patients. This could be one of the reasons why the item did not work well in the model and why it did in pre-pandemic studies. One reason from a methodological point of view is that it is the only item in the whole PD dimension, and the whole test, that contains an explicit negation (not), which may have given problems in the comprehension of the item when it was answered.”*

*Observation 3: *The suggestion was added to the manuscript: *“Despite the above, it was decided to keep the item, as its content is not interchangeable with any other item in the PD dimension, and removing it would imply an under-representation of content in the dimension. Moreover, it would limit the possibility of directly comparing the results obtained here with previous studies.”*

*Observation 4: *The suggestion was added to the manuscript: *“In the bifactor model, convergence with subsequent modifications (e.g. deletion of items) was not assessed to avoid capitalization of chance and possible under-representation of dimension content.”*

# Reviewer report: Reviewer response for version 1

**DOI:** 10.5256/f1000research.152521.r221619

**Published:** 2023-12-08

**Authors:** Arnold Alejandro Tafur Mendoza

**Affiliations:** 1 Universidad Peruana de Ciencias Aplicadas, Lima District, Lima Region, Peru

The article presents the evaluation of the psychometric properties of one of the most widely used scales for the measurement of burnout syndrome, the Maslach Burnout Inventory (MBI), in a relevant sample such as health professionals who, according to the existing literature, is the main group that suffers the negative consequences of burnout (stress, anxiety, etc.). The article is well structured and reports the results of analyses relevant to the functioning of the MBI, exploring various sources of validity evidence (based on content, internal structure and relationship with other variables), as well as the consideration of the omega coefficient for the analysis of the reliability of the scores. Furthermore, the results obtained are adequately discussed in contrast with the relevant and updated literature, pointing out the limitations and contribution of the research.

 In order to improve the reporting of the research conducted, I would recommend the following minor corrections to clarify some of the terms used in the article.

1. Remove Table 5 or Figure 1 from the text, as both elements contain the same information and are therefore redundant. In particular, I would remove Table 5, as the visualisation of the results of the confirmatory factor analysis (the correlation between the factors, the factor loadings and the error variances) could be clearer for the reader.
2. In Table 2, replace DE, As and Cu by SD, Sk and Ku, as the former are used because they are in Spanish and the latter correspond to their English version. The article is in English. The same happens in Table 7, where "Asymmetry" should be replaced by "Skewness", which is the correct statistical term in English. In Table 3, instead of "Average" there should be "Mean". In Table 4, instead of "IC" there should be "CI", which corresponds to the confidence interval. Finally, in Table 6 there are again problems with some of the statistics mentioned above. It would be important to review the entire article again to verify that there is no other error with the English language.
3. On page 3, in the study by González-Rodríguez et al. (2022), it is necessary to indicate how they evaluated the evidence of convergent validity and what results they obtained. Also, indicate which structure they evaluated, since they indicate that it was the original model (I understand the three-factor model), but then they indicate that they found four factors. Clarify the reporting of these results.
4. On page 6, clarify the wording of the objectives. In objective 1, it is incorrect to say that validity is established, what is done is to provide evidence of validity, where appropriate, based on the content of the test through the criteria of expert judges and based on the internal structure through confirmatory factor analysis. In objective 2, a more appropriate term is "Estimate reliability" and if appropriate also add that they used the omega coefficient. In objective 3, it is better to indicate that they sought to provide evidence of validity based on the relationship with other variables and to indicate the methods they used for convergent (AVE) and discriminant (HTMT2 ratio and Fornell and Larcker) evidence, as stated on page 7. In objective 4 (and on page 7), there is an error, as they did not use ROC curves for interpretive norms, but percentile scores. Remove objective 5, as it looks more like a hypothesis and is not relevant for this type of psychometric study.
5. On page 7, it should be added that the omega coefficient was also estimated for the assessment of internal consistency reliability.
6. Delete the last paragraph on page 7, as it is redundant with what is already mentioned at the top of the page. End this section only by pointing out everything related to the software used.

With these minor corrections, the article would be clearer for the interested reader. Success in the process of reviewing the comments.

Is the work clearly and accurately presented and does it cite the current literature?

Yes

If applicable, is the statistical analysis and its interpretation appropriate?

Yes

Are all the source data underlying the results available to ensure full reproducibility?

Yes

Is the study design appropriate and is the work technically sound?

Yes

Are the conclusions drawn adequately supported by the results?

Yes

Are sufficient details of methods and analysis provided to allow replication by others?

Yes

Reviewer Expertise:

psychometrics, measurement, assessment, quantitative methodology, factor analysis.

I confirm that I have read this submission and believe that I have an appropriate level of expertise to confirm that it is of an acceptable scientific standard.

# Response

**Published:** 2024-02-20

**Authors:** Enaidy Reynosa Navarro

**Affiliations:** Universidad César Vallejo, Peru

Observation 1: The suggestion was added, and Table 5 was deleted.

 Observation 2: The indicated corrections were made and the full text was checked for any other English language errors. The indicated corrections were made and the full text was checked for any other English language errors.

 Observation 3: The suggestion was added: *“Results indicated an acceptable fit for the four-factor correlated model (Emotional Exhaustion, Depersonalization, Personal Realization, and Interpersonal Psychological Stress). Cronbach's alpha coefficients indicated acceptable reliability for all four factors, with values between .718 and .911 (31).”*

 Observation 4: The objectives were modified as suggested:

*1. Provide valid evidence of the MBI in healthcare professionals from the Ancash region, Peru, based on the content of the test through the criteria of expert judges and based on the internal structure through confirmatory factor analysis (CFA).*

*2. Estimate the reliability of the MBI in healthcare professionals from the Ancash region, Peru, using Cronbach's alpha and omega coefficient.*

*3. Provide evidence of validity based on the relationship with other variables through convergent (average variance extracted, AVE) and discriminant evidence (HTMT2 ratio and the Fornell and Larcker criterion).*

*4. To compute MBI scores to obtain interpretative norms (reference values) for the instrument in healthcare professionals from the Ancash region, Peru, using percentile scores.*

 Observation 5: The suggestion was added to the manuscript: *“…and omega coefficient. These coefficients range from 0 to 1…”*

 Observation 6: The paragraph was deleted: *“In the current study, meticulous steps were undertaken to counteract and mitigate possible biases. Before the survey's distribution, approval was secured from the leadership of the involved hospitals. Participation was restricted to those healthcare professionals who provided their informed consent. To ensure a neutral selection process, invitations were disseminated to potential participants via WhatsApp and email. Furthermore, any incomplete responses were eliminated during the data refinement phase, safeguarding the integrity and accuracy of the concluding data.”*
